# Vardenafil Alleviates Doxorubicin-Induced Cardiotoxicity Associated with Restoration of the AMPK/SIRT1 Signaling Pathway

**DOI:** 10.3390/biomedicines14081721

**Published:** 2026-07-31

**Authors:** Eman H. Yousef, Mohamad A. El-Gammal, Muhammed M. Salahuddin, Mahmoud Abdelbadie Salem, Amal Abdullah Alrashidi, Ahmed G. Abd Elhameed

**Affiliations:** 1 Department of Pharmacology and Biochemistry (Biochemistry), Faculty of Pharmacy, Horus University-Egypt, New Damietta 34518, Egypt; ehamdy@horus.edu.eg (E.H.Y.); msalahuddin@horus.edu.eg (M.M.S.); 2 Department of Pharmacology and Biochemistry (Pharmacology), Faculty of Pharmacy, Horus University-Egypt, New Damietta 34518, Egypt; melgamal@horus.edu.eg (M.A.E.-G.); ahmed_gamal_helal@mans.edu.eg (A.G.A.E.); 3 Cardiovascular Medicine Department, Faculty of Medicine, Mansoura University, Mansoura 35516, Egypt; mahmoudbadie@mans.edu.eg; 4 Pharmaceutical Sciences Department, College of Pharmacy, Princess Nourah Bint Abdullarhman University, Riyadh 11671, Saudi Arabia; 5 Pharmacology and Toxicology Department, Faculty of Pharmacy, Mansoura University, Mansoura 35516, Egypt

**Keywords:** doxorubicin, phosphodiesterase, cardiotoxicity, AMPK, deacetylase, oxidative stress, apoptosis

## Abstract

**Background**: Doxorubicin-induced cardiotoxicity (DIC) is a major limitation of anthracycline chemotherapy and is characterized by oxidative stress, apoptosis, and myocardial remodeling. Dysregulation of AMP-activated protein kinase (AMPK) and its downstream effector sirtuin-1 (SIRT1) has been implicated in the molecular pathogenesis of DIC. Vardenafil (Var), a selective phosphodiesterase-5 inhibitor, has shown cardiovascular benefits; however, its impact on AMPK/SIRT1 signaling during DIC remains unclear. **Methods**: DIC was induced in rats by cumulative doxorubicin administration. Cardiac function, serum injury biomarkers, oxidative stress indices, histopathological alterations, apoptosis, fibrosis, and molecular markers associated with the AMPK/SIRT1 pathway were evaluated. In addition, molecular docking was performed to assess the potential interaction of Var with AMPK. **Results**: Var significantly improved cardiac function and reduced serum levels of lactate dehydrogenase, creatine kinase, and blood urea nitrogen. Treatment attenuated myocardial oxidative stress, restored glutathione content, reduced lipid peroxidation, and alleviated histopathological damage. Furthermore, Var suppressed caspase-3 and TGF-β1 expression while enhancing HO-1 levels. Var treatment was associated with restoration of cardiac phosphorylated AMPK (p-AMPK) expression and increased SIRT1, Nrf2, and PPARγ levels in doxorubicin-treated rats, consistent with modulation of an AMPK/SIRT1-associated cytoprotective network. Molecular docking demonstrated favorable interactions between Var and AMPK, providing supportive in silico evidence for the observed molecular findings. **Conclusions**: These findings demonstrate that Var attenuates doxorubicin-induced cardiotoxicity, and its cardioprotective effects are associated with restoration of the p-AMPK/SIRT1/Nrf2/PPARγ signaling pathway, together with reductions in oxidative stress, apoptosis, and fibrosis. Collectively, these findings suggest that Var attenuates early Dox-induced cardiac injury, potentially through modulation of the AMPK/SIRT1 signaling pathway, warranting further validation in long-term and dose–response studies.

## 1. Introduction

Doxorubicin (Dox), an anthracycline chemotherapy, continues to be the mainstay in the treatment of various cancers, including cervical cancer, leukemia, lung cancer, breast cancer, and ovarian cancer in clinical use. However, its clinical application and therapeutic potential are strongly hampered by serious cardiotoxic effects, which often result in congestive heart failure and, ultimately, cardiac death [1,2,3,4]. The pathogenesis of Dox-induced heart toxicity involves several factors, mainly oxidative stress, inflammation, and apoptosis [5,6]. Consequently, prophylactic cardio-protection offers distinct advantages in mitigating the adverse myocardial effects of Dox compared to treatments initiated post-cardiotoxicity onset [7]. Carvedilol (Carv) has been extensively documented for its cardioprotective efficacy in patients undergoing anthracycline chemotherapy [8,9]; however, its use is associated with side effects such as erectile dysfunction. Moreover, Carv is contraindicated in patients with uncontrolled bronchospastic disease [10].

The 5′-Adenosine monophosphate (AMP)-activated protein kinase (AMPK) is a highly conserved eukaryotic protein serine/threonine kinase, playing a vital role in maintaining both cellular and systemic energy homeostasis. It facilitates various processes essential for energy and redox balance, such as autophagy, mitochondrial biogenesis, and the regulation of glucose and lipid metabolism [11]. AMPK is highly expressed in the heart [12] and is AMP/ATP ratio-sensitive. Therefore, every cellular activity that either raises AMP concentrations or depletes ATP levels activates it [13]. Once activated, AMPK can stimulate the silent information regulator 1 (SIRT1) by increasing the cellular NAD^+^/NADH ratio, leading to deacetylation of downstream SIRT1 targets and modulation of their activity [14,15]. SIRT1, an NAD^+^-dependent deacetylase, is involved in various biological responses, including aging, oncogenesis, glucose metabolism, and energy regulation. SIRT1 has demonstrated efficacy in alleviating oxidative stress, inflammation, cellular senescence, and programmed cell death [16]. In the myocardium, SIRT1 is crucial for normal cardiomyocyte development, as evidenced by the presence of cardiac malformations in Sirt1 knockout mice [17]. Evidence has indicated that Dox-induced cardiotoxicity significantly inhibits AMPK activity and downregulates SIRT1 expression [18,19,20]. Meanwhile, the activation of AMPK has been shown to alleviate Dox-induced cardiac damage and dysfunction [21,22]. Overall, these observations indicate that targeting AMPK could be a promising approach to prevent Dox-induced cardiotoxicity.

Vardenafil (Var) is a powerful and highly selective inhibitor of phosphodiesterase-5 (PDE-5), used in the therapeutic management of erectile dysfunction and pulmonary arterial hypertension [23,24]. PDE-5 is responsible for the hydrolysis of cyclic guanosine monophosphate (cGMP) within smooth muscle cells, and inhibition of PDE-5 results in vasodilation of the corpus cavernosum and pulmonary vasculature driven by nitric oxide-mediated accumulation of cGMP [24]. Continuous inhibition of PDE-5 has been demonstrated to downregulate vascular inflammatory protein expression, hence enhancing tissue protection [25,26]. In addition to those effects, Var has been reported to elevate the activity of antioxidant enzymes, including superoxide dismutase and catalase [27]. It also exhibits protective properties by reducing oxidative stress and suppressing nuclear factor kappa B (NF-κB) activation in experimental models of hepatitis and cystic fibrosis [28,29]. Furthermore, Var treatment has been found to protect against lithocholic acid (LCA)-induced cholestatic liver injury by activating the Nrf2 signaling pathway and inhibiting the activation of the NLRP3 inflammasome [30]. Nevertheless, data on the impact of Var pre-treatment on Dox-induced cardiac toxicity is limited, and the precise molecular mechanisms have yet to be fully elucidated.

Given the evidence implicating AMPK/SIRT1 signaling in the regulation of oxidative stress, apoptosis, and tissue remodeling during cardiac injury, we hypothesized that Var may attenuate Dox-induced cardiotoxicity through modulation of this molecular network. Therefore, the present study was designed to investigate the cardioprotective effects of Var in an experimental model of Dox-induced cardiotoxicity and to elucidate the potential involvement of the AMPK/SIRT1 pathway and its downstream redox-, apoptotic-, and fibrosis-related targets.

## 2. Materials and Methods

### 2.1. Molecular Docking of Var to AMPK

The three-dimensional structure of Var was obtained from the ZINC database (https://zinc.docking.org/), while the crystal structure of AMPK (PDB ID: 4zhx) was downloaded from the Protein Data Bank (https://www.rcsb.org/). Both Var and AMPK structures were converted to pdbqt format using AutoDockTools (version 1.5.6, http://autodock.scripps.edu/). Molecular docking was then carried out using AutoDock Vina 1.2.0. The resulting binding interactions and conformations within the active site were examined and visualized using Chimera 1.15 and Discovery Studio Visualizer v21.1.0.20298.

### 2.2. Drugs and Chemicals

Dox, Carv and Var were supplied as pharmaceutical drugs (for Dox, Adriamycin^®^ vial, 50 mg/25 mL, Pfizer Inc., New York, NY, USA; for Carv, Carvipress^®^ tablet, 50 mg, Global Napi Pharmaceuticals, Giza, Egypt; and for Var, Powerecta^®^ disintegrating tablet, 10 mg, EVA PHARMA, Cairo, Egypt). Carv and Var were prepared for oral administration by grinding them into fine powder and suspending them in 1% (*w*/*v*) carboxymethyl cellulose (CMC) solution. Urethane and other reagents used in the experiments were procured from Sigma-Aldrich Chemical Co. (St. Louis, MO, USA).

### 2.3. Animals

Twenty adult male albino Wistar rats, each weighing approximately 200 ± 50 g, were procured from the Egyptian Organization for Biological Products and Vaccines (Vacsera, Giza, Egypt). Throughout the study period, the animals were housed under standard laboratory conditions, including a 12 h light/dark cycle, a controlled temperature of 25 ± 3 °C, and a relative humidity of 30–70%. They were also given unrestricted access to food and water *ad* libitum. Prior to the initiation of the experiment, all rats were acclimatized for one week. All animal experiments were conducted in accordance with the ARRIVE (Animal Research: Reporting of In Vivo Experiments) guidelines and the National Institutes of Health (NIH) Guide for the Care and Use of Laboratory Animals (NIH Publication No. 85–23, revised 2011). The study protocol was reviewed and approved by the Research Ethics Committee of Horus University—Egypt on 9 November 2025 (Approval No. PH-2025-003). All efforts were made to minimize animal suffering and to reduce the number of animals used.

### 2.4. Induction of Doxorubicin Cardiotoxicity

Cardiotoxicity was induced following a previously established model of cardiotoxicity study design [31]. Dox was administered intraperitoneally at a dose of 2.5 mg/kg, given in six equal injections on alternate days beginning in the third week of the study, resulting in a total cumulative dose of 15 mg/kg [32].

### 2.5. Experimental Design

**Figure 1:**
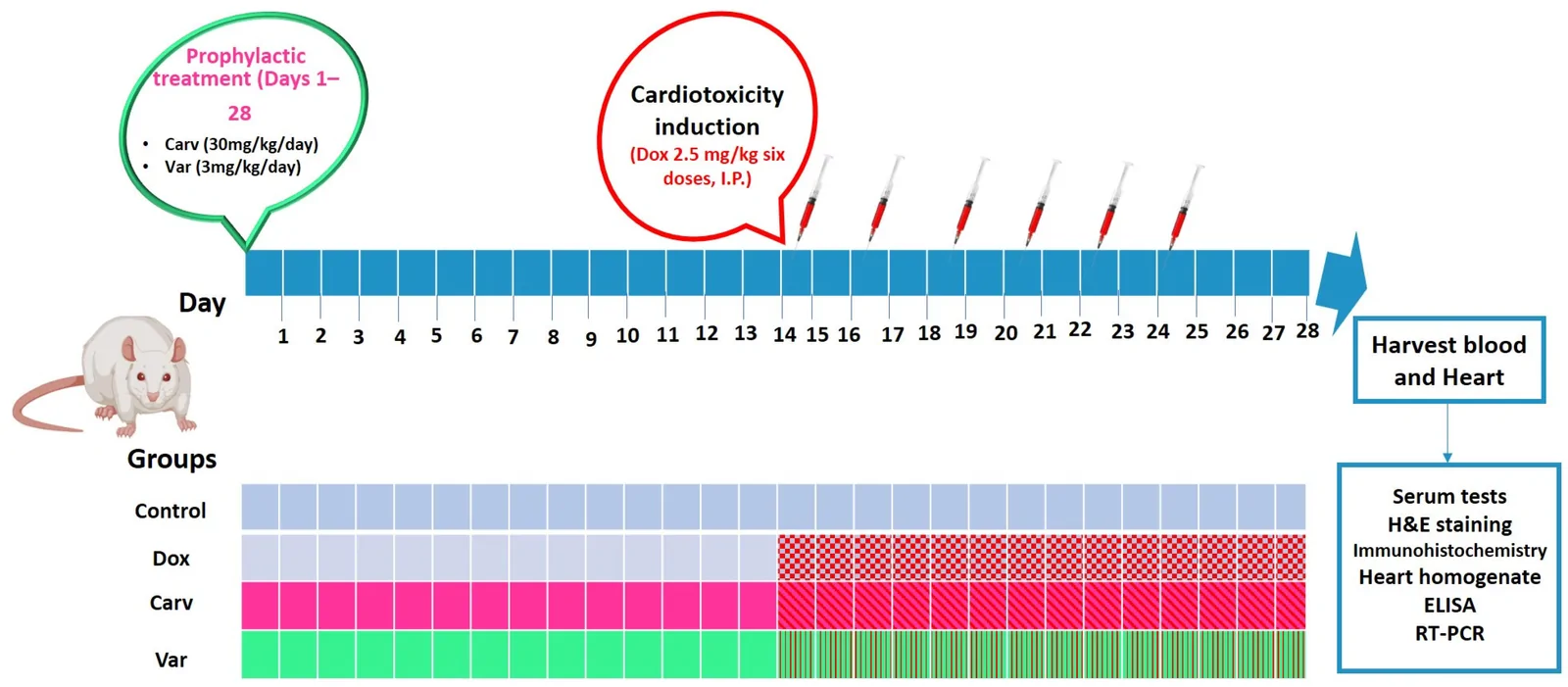
Experimental design of the prophylactic treatment protocol. Rats received oral vardenafil (Var, 3 mg/kg/day) or carvedilol (Carv, 30 mg/kg/day) once daily for 28 consecutive days, with treatment initiated 14 days before the first doxorubicin (Dox) injection and continued throughout the Dox administration period. Dox-induced cardiotoxicity was established by six intraperitoneal injections of Dox (2.5 mg/kg each) administered on alternate days from Day 14 to Day 24. On Day 28, animals were sacrificed, and blood and heart tissues were collected for serum biochemical, histopathological, immunohistochemical, ELISA, and qRT-PCR analyses.

The animals were randomly allocated into four experimental groups (Figure 1) (*n* = 6 per group) as follows:

- Group I (Control): Rats administered an oral dose of 1% *w*/*v* CMC aqueous solution for the duration of 4 weeks.
- Group II (Dox): Rats underwent induction of cardiotoxicity as described in the previous section.
- Group III (Carv): Rats underwent induction of cardiotoxicity as described in the previous section. Carv (30 mg/kg/day, orally, suspended in 3% *w*/*v* CMC solution, 1 mL/kg) was administered prophylactically for the entire 4-week experimental period, beginning 14 days before the first Dox injection and continuing throughout the Dox administration period [33]. Carv was used as the standard cardioprotective drug in the DIC model.
- Group IV (Var): Rats underwent induction of cardiotoxicity as described in the previous section. Vardenafil (3 mg/kg/day, orally, suspended in 3% *w*/*v* CMC solution, 1 mL/kg) was administered prophylactically for the entire 4-week experimental period, beginning 14 days before the first Dox injection and continuing throughout the Dox administration period [34].

A statistical power analysis was conducted to determine sample size with the following parameters: effect size = 0.8, α error = 0.05, power = 0.8, and number of groups = 4, which was also in line with the sample size used in the previous study with the same animal model [35].

### 2.6. Biological Sample Collection

At the end of the experiment, animals were fasted for 12 h overnight before sacrifice while maintaining free access to water to minimize dehydration, standardize their metabolic status and minimize inter-animal variability. Subsequently, the rats were anesthetized using urethane (1.8 g/kg, intraperitoneally) [36] and echocardiography was performed. Blood samples were subsequently collected from the orbital sinus prior to euthanasia by decapitation. The blood was left to clot for 30 min at 25 °C and then centrifuged at 2000 *g* for 10 min to separate the serum, which was stored at −80 °C for further analysis. The hearts were excised, rinsed with physiological saline containing heparin (0.16 mg/mL) to prevent coagulation, blotted dry with filter paper, and weighed. Each heart was divided into three sections: the lower portion (0.5 g of the cardiac apex) was fixed in 10% (*v*/*v*) neutral buffered formalin for 24 h for histopathological evaluation; the second portion was immediately frozen in liquid nitrogen for quantitative real-time PCR (qRT-PCR) analysis; and the third portion was homogenized using an Omni-125 portable homogenizer (Omni International, Kennesaw, GA, USA) in 10% *w*/*v* 50 mM K_2_HPO_4_ buffer (pH 7.5). The homogenates were centrifuged at 3000 *g* for 20 min at 4 °C using a cooling centrifuge (Damon/IEC Division, Model CRU-5000, Needham, MA, USA). The resulting supernatants were collected and kept on ice for subsequent analyses of MDA, GSH, and ELISA assays.

### 2.7. Echocardiography

Twenty-eight days after initiating the treatment protocol, and prior to sacrificing the animals, cardiac function was evaluated by echocardiography. For this procedure, rats were anesthetized with urethane (1.8 g/kg, intraperitoneally), and their chest area was shaved before being placed in a supine position. Two-dimensional transthoracic echocardiography was performed using the M5Sc transducer connected to GE Vivid E9 XDclear Dimensions ultrasound system (GE Healthcare, Chicago, IL, USA). The examination was conducted by an expert echocardiographer who was blinded regarding group assignments. The GE 6S-RS cardiac sector probe, applied with ultrasound gel, was positioned at the left parasternal area. Echocardiographic measurements and image analyses were also performed in a blinded manner, with the investigator analyzing the recordings unaware of the treatment allocation. The acquired images were analyzed to assess left ventricular function, including end-diastolic diameter (IVSd), end-systolic diameter (IVSs), left ventricular internal diameter at end-diastole (LVIDd), left ventricular internal diameter at end-systole (LVIDs), ejection fraction (%EF), and fractional shortening (%FS), which were calculated as the mean of three consecutive cardiac cycles.

### 2.8. Biochemical Evaluation

Serum concentrations of cardiac injury indicators, including creatine kinase (CK), creatine kinase-MB (CK-MB), and lactate dehydrogenase (LDH), along with serum creatinine (Scr) and blood urea nitrogen (BUN), were measured using commercially supplied assay kits (Biodiagnostic, Giza, Egypt). Furthermore, cardiac tissue homogenates were analyzed for the antioxidant reduced glutathione (GSH) and the lipid peroxidation marker malondialdehyde (MDA) using locally available commercial kits (Biodiagnostic, Egypt).

### 2.9. Histopathological and Immunohistochemical Analysis

Routine histological staining (H&E) was used to identify histological alterations in the cardiac tissue. Formalin-fixed heart samples were embedded in paraffin, sectioned at a thickness of 5 µm, and subsequently stained. Images were captured using a digital camera attached to a BX51 Olympus optical microscope (Olympus Corporation, Tokyo, Japan).

Monoclonal antibodies for caspase-3 (Cat. No. A11319; ABclonal Co., Woburn, MA, USA), TGF-β1 (Cat. No. A2407; ABclonal Co.), and HO-1 (Cat. No. A1346; ABclonal Co.) were used in immunohistochemical studies on 5-μm deparaffinized heart sections that had been incubated overnight at 4 °C. The sections were then treated with horseradish peroxidase-conjugated secondary antibodies (Abcam, Cambridge, UK). Visualization was achieved using 2% diaminobenzidine prepared in 50 mM Tris buffer (pH 7.6). ImageJ software was used to quantify immunostained regions that showed brown coloring (version 1.53c; National Institutes of Health, Bethesda, MD, USA). Digital image analysis was standardized by setting a 25 µm scale bar and adjusting image dimensions to 12.7 cm × 9 cm at 300 dpi. The positive area % was calculated. Immunohistochemical image acquisition, quantification, and scoring were performed by an investigator blinded to the treatment group allocation.

### 2.10. Western Blot Assay

Western blot analysis was carried out to determine the protein expression levels in cardiac tissues. Total proteins were isolated following the manufacturer’s instructions of the total protein extraction kit, and their concentrations were measured using the BCA Protein Assay. Equal amounts of protein samples were loaded onto 8% sodium dodecyl sulfate–polyacrylamide gels (SDS-PAGE) and subsequently transferred to nitrocellulose membranes. The membranes were blocked with 5% non-fat milk for 1.5 h and then incubated overnight at 4 °C with the primary antibodies against p-AMPK (Thr172) (rabbit polyclonal, Cat. No. PA5-17831, Thermo Fisher Scientific, Waltham, MA, USA; 1:500) and β-actin (mouse monoclonal, clone 15G5A11/E2, Cat. No. MA1-140, Thermo Fisher Scientific, Waltham, MA, USA; 1:5000) as a loading control. After washing, the membranes were incubated for 2 h at room temperature with the HRP-conjugated secondary antibody. Protein bands were visualized using the Odyssey Infrared Imaging System (LI-COR, Lincoln, NE, USA) and quantified using ImageJ software (NIH, Bethesda, MD, USA).

### 2.11. ELISA Assay

Cardiac protein concentrations of SIRT1, nuclear factor erythroid 2–related factor 2 (Nrf2), p-AMPK, and peroxisome proliferator-activated receptor gamma (PPARγ) were quantified in supernatant from heart tissue homogenates using ELISA kits following the manufacturer’s protocol. SIRT1 and Nrf2 were measured using ELISA kits obtained from MyBioSource (San Diego, CA, USA), whereas p-AMPK was measured using AMPK ELISA Kit (SunLong Biotech Co., Ltd., Hangzhou, Zhejiang, China; Cat. No. SL0570Ra) and PPARγ was quantified using PPARγ ELISA Kit (FineTest, Wuhan Fine Biotech Co., Ltd., Wuhan, China; Cat. No. ER1284). These markers were assessed at the protein level and were therefore not included in the qRT-PCR primer table.

### 2.12. Quantitative Real-Time Polymerase Chain Reaction (qRT-PCR)

Quantitative real-time polymerase chain reaction (qRT-PCR) was employed to assess the expression of the cardiac *PPARγ* gene. Total RNA was extracted from heart tissue using TRIzol reagent (Thermo Fisher Scientific, Waltham, MA, USA). The yield and purity of the isolated RNA were determined spectrophotometrically at 260 nm and by calculating the 260/280 nm absorbance ratio with a NanoDrop spectrophotometer (Thermo Fisher Scientific Inc., Houston, TX, USA). Only RNA samples with purity ratios ≥1.8 were used for subsequent analysis. Complementary DNA (cDNA) was synthesized from 1 µg of total RNA using the Qiagen RT-PCR kit (Qiagen, Beverly, MA, USA). qRT-PCR amplification was then conducted with Maxima SYBR Green/Fluorescein qPCR Master Mix (Thermo Scientific, USA) on a Rotor-Gene Q thermocycler (Qiagen, USA). Each 25 µL reaction mixture contained 12.5 µL of SYBR^®^ Green PCR Master Mix, 2 µL of forward and reverse primers, 5.5 µL of RNase/DNase-free water, and 3 µL of cDNA. The mixture was gently vortexed and briefly centrifuged before amplification. Thermal cycling conditions were as follows: an initial activation step at 95 °C for 10 min, followed by 45 cycles consisting of denaturation at 95 °C for 10 s, annealing at 60 °C for 15 s, and extension at 72 °C for 15 s. Primer specificity was verified through melt-curve analysis. Gene-specific primers were designed using the PrimerQuest Tool (https://www.idtdna.com/PrimerQuest, accessed on 13 December 2025) based on sequences obtained from PubMed (Entrez Gene) and synthesized by Invitrogen Co. (Carlsbad, CA, USA). Expression levels of the target gene were normalized to the housekeeping gene β-actin, and relative gene expression was calculated using the 2^−ΔΔCT^ method. Only PPARγ gene expression was quantified by qRT-PCR using β-actin as the endogenous reference gene. Accordingly, primer sequences are provided only for PPARγ and β-actin (Table 1).

### 2.13. Statistical Analysis

Data processing and graphical visualization were performed using GraphPad Prism version 6.01 (GraphPad Software, San Diego, CA, USA). Data were assessed for normality using the Shapiro–Wilk test and for homogeneity of variances using Levene’s test. Normally distributed data with homogeneous variances were analyzed using one-way analysis of variance (ANOVA) followed by Tukey’s multiple-comparisons post hoc test. Immunohistochemical scores were analyzed using the Kruskal–Wallis test followed by Dunn’s multiple-comparisons test. Results are expressed as mean ± standard deviation (SD), and a two-tailed *p* value < 0.05 was considered statistically significant. Unless otherwise stated, the sample size (*n*) represents the number of experimental animals (biological replicates) in each group. Western blot analysis was performed using three independent biological replicates per group (*n* = 3), whereas all other quantitative analyses were performed using six biological replicates per group (*n* = 6).

## 3. Results

### 3.1. Var Protected Against Dox-Induced Myocardial Injury in DIC Rat Model

**Figure 2:**
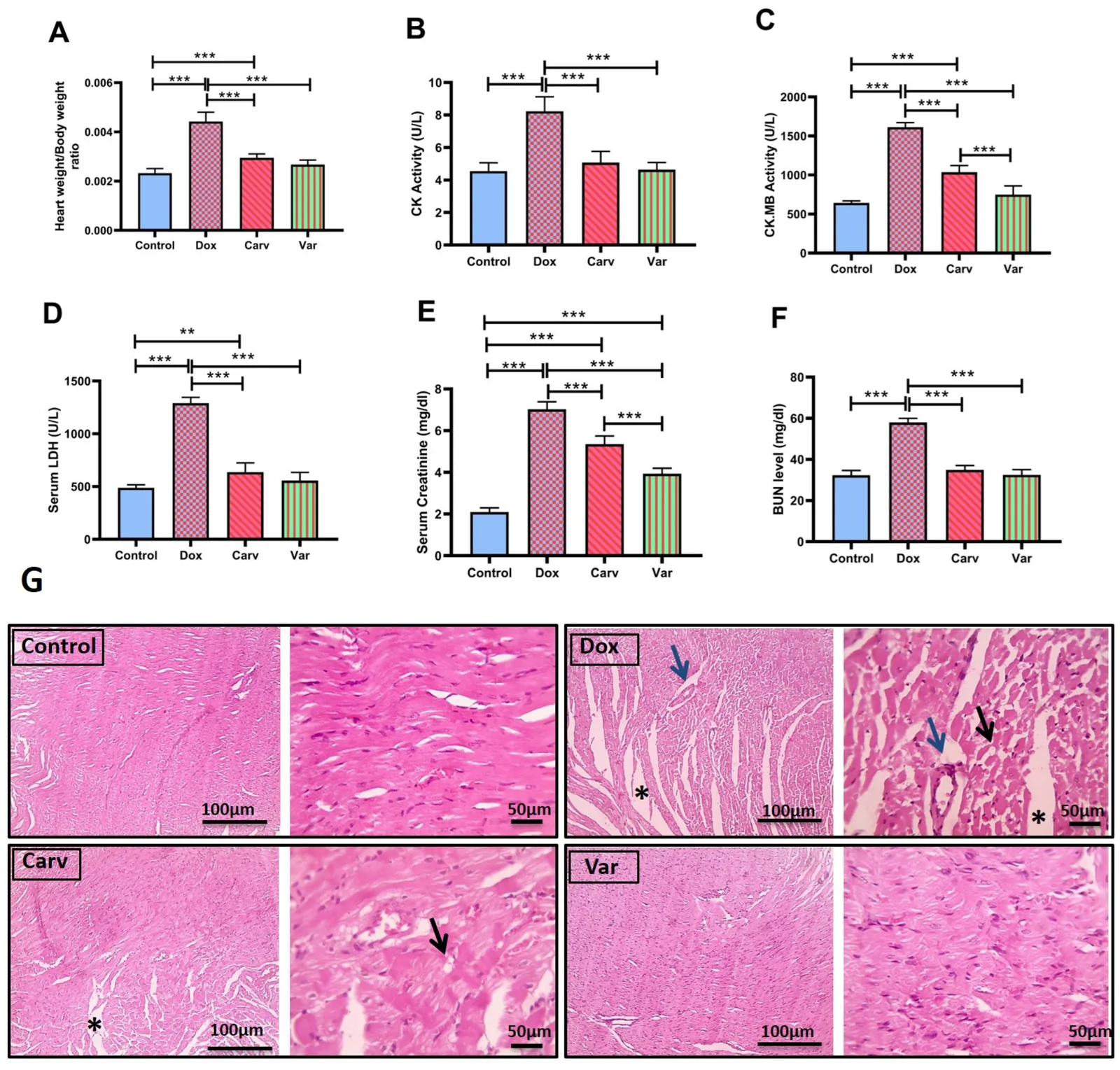
Var protected against Dox-induced myocardial injury in the DIC rat model. Effect of Var against DIC was evaluated in the different groups by (**A**) heart/body weight ratio, (**B**) serum creatine kinase (CK) activity (U/L), (**C**) serum creatine kinase MB (CK-MB) activity (U/L), (**D**) serum lactate dehydrogenase (LDH) activity (U/L), (**E**) serum creatinine level (mg/dL), (**F**) blood urea nitrogen (BUN) level (mg/dL), and (**G**) histopathological investigation of heart tissue stained by H&E. * indicates prominent interstitial, blue arrow indicates perivascular edema, and black arrows indicate marked vacuolation of muscle fibers. Magnifications ×100: bar 100 and ×400: bar 50. Data are presented as mean ± SD (*n* = 6 biological replicates per group, where each biological replicate represents one animal). Statistical significance denoted as ** *p*  ≤  0.01, and *** *p*  ≤  0.001.

To investigate the cardioprotective potential of Var against Dox-induced myocardial injury, an acute model of DIC was established. The effects of Var on cardiac morphology and biochemical parameters were then evaluated. A marked increase in the heart weight-to-body weight ratio was observed in the Dox-treated group compared with the control group, indicating cardiac hypertrophy. However, treatment with either Carv or Var effectively mitigated this Dox-induced elevation (Figure 2A).

Myocardial damage was further evaluated by measuring serum CK-MB, CK activities, and LDH levels. Dox administration caused significant elevations in CK-MB (2.5-fold; F (3, 20) = 189.3, *p* < 0.0001, *n* = 6), CK (1.81-fold; F (3, 20) = 43.32, *p* < 0.0001, *n* = 6), and LDH (2.64-fold; F (3, 20) = 188.2, *p* < 0.0001, *n* = 6) relative to the control group. In contrast, Carv treatment reduced these parameters by 35.83%, 38.43%, and 50.59%, respectively, compared with the Dox group. Similarly, Var administration lowered CK-MB, CK, and LDH levels by 53.51%, 43.69%, and 56.87%, respectively, compared with Dox alone. Also, Var administration reduced CK-MB level by 27.6% (F (3, 20) = 189.3, *p* < 0.001, *n* = 6) as compared with Carv. These findings suggest that both Carv and Var significantly attenuate Dox-induced myocardial injury, with Var demonstrating a cardioprotective effect comparable to that of Carv (Figure 2B–D).

Dox-induced cardiotoxicity has been linked to a pronounced decline in cardiac output [37]. A reduction in cardiac output (CO) consequently results in a disproportionately greater decrease in renal perfusion, leading to a subsequent drop in the glomerular filtration rate (GFR) [38]. In line with these pathophysiological effects, our findings revealed that Dox treatment significantly elevated blood urea nitrogen (BUN) and serum creatinine levels by 1.79-fold (F (3, 20) = 184.3, *p* < 0.0001, *n* = 6) and 3.36-fold (F (3, 20) = 275.9, *p* < 0.0001, *n* = 6), respectively, compared with the control group. The marked increases in BUN and serum creatinine in the Dox group are likely attributed to both the nephrotoxic and cardiotoxic consequences of Dox exposure. Conversely, treatment with Carv attenuated these elevations, reducing BUN and serum creatinine levels by 39.87% (F (3, 20) = 184.3, *p* < 0.0001, *n* = 6) and 23.88% (F (3, 20) = 275.9, *p* < 0.0001, *n* = 6), respectively, relative to the Dox group. Likewise, Var treatment resulted in 43.9% (F (3, 20) = 184.3, *p* < 0.0001, *n* = 6) and 44.04% (F (3, 20) = 275.9, *p* < 0.0001, *n* = 6) reductions in BUN and serum creatinine levels, respectively, compared with the Dox group. In addition, Var treatment caused a reduction in serum creatinine level by 26.5% (F (3, 20) = 275.9, *p* < 0.001, *n* = 6) as compared with Carv (Figure 2E,F).

H&E staining was used to assess cardiac tissue structure. In the H&E staining, we also observed that there was prominent interstitial and perivascular edema and marked vacuolation of muscle fibers in the Dox group, while this change was less noticeable in the Carv group. Also, cardiac sections from the Var group showed a retained normal histological picture of muscle fibers, blood vessels and interstitial tissue. Overall, our findings showed that Var reduced the in vivo cardiac dysfunction caused by Dox (Figure 2G).

**Figure 3:**
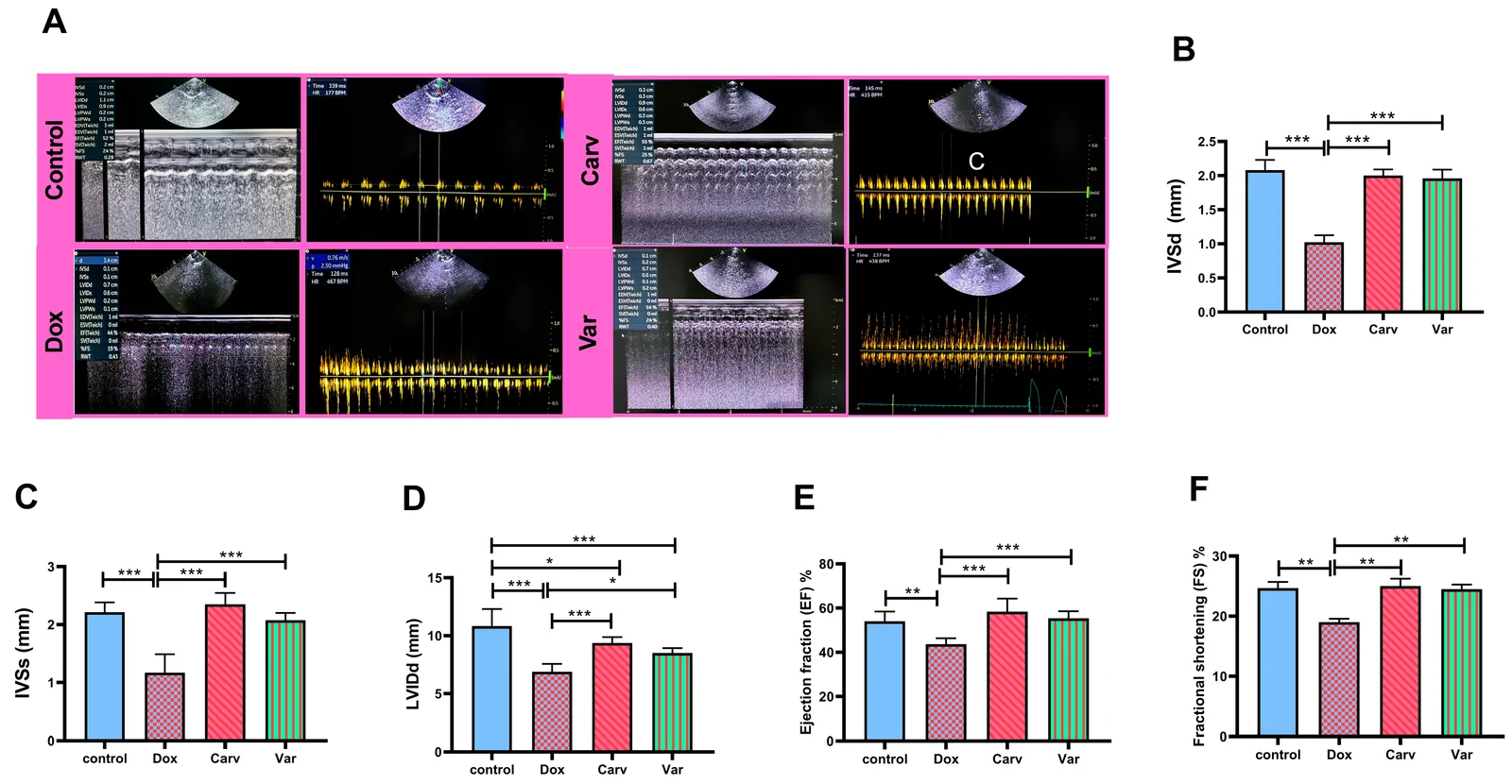
Vardenafil improves left ventricular function in Dox-induced cardiotoxicity. (**A**) Echocardiography was performed at day 28 after initiating the treatment protocol; (**B**) IVSd: interventricular septal thickness in diastole; (**C**) IVSs: interventricular septal thickness in systole; (**D**) LVIDd: left ventricular internal diameter at end-diastole; (**E**) EF: ejection fraction; (**F**) FS: fractional shortening. Data are presented as mean ± SD (*n* = 6 biological replicates per group, where each biological replicate represents one animal). Statistical significance denoted as * *p*  < 0.05, ** *p*  ≤  0.01, and *** *p*  ≤  0.001.

Dox administration resulted in a marked deterioration of left ventricular systolic performance, as evidenced by significant reduction in IVSd, IVSs, LVIDd, EF%, and FS% by 2.0 (*p* < 0.001), 1.89 (*p* < 0.001), 1.57 (*p* < 0.001), 1.2 (*p* < 0.01), and 1.3-fold (*p* < 0.01), respectively, compared with the control group. Treatment with either Carv or Var significantly attenuated these changes, demonstrating preservation of systolic function. Relative to the Dox group, IVSd was increased by 1.95- and 1.91-fold in the Carv and Var groups, respectively. Similarly, IVSs increased by 2.0- and 1.77-fold, while LVIDd increased by 1.3- and 1.2-fold, EF% by 1.33- and 1.26-fold, and FS% by 1.3- and 1.28-fold, respectively. These results indicate the capacity of both Carv and Var to mitigate Dox-induced systolic dysfunction, with comparable cardioprotective efficacy (Figure 3).

### 3.2. Var Modulated AMPK Activity

**Figure 4:**
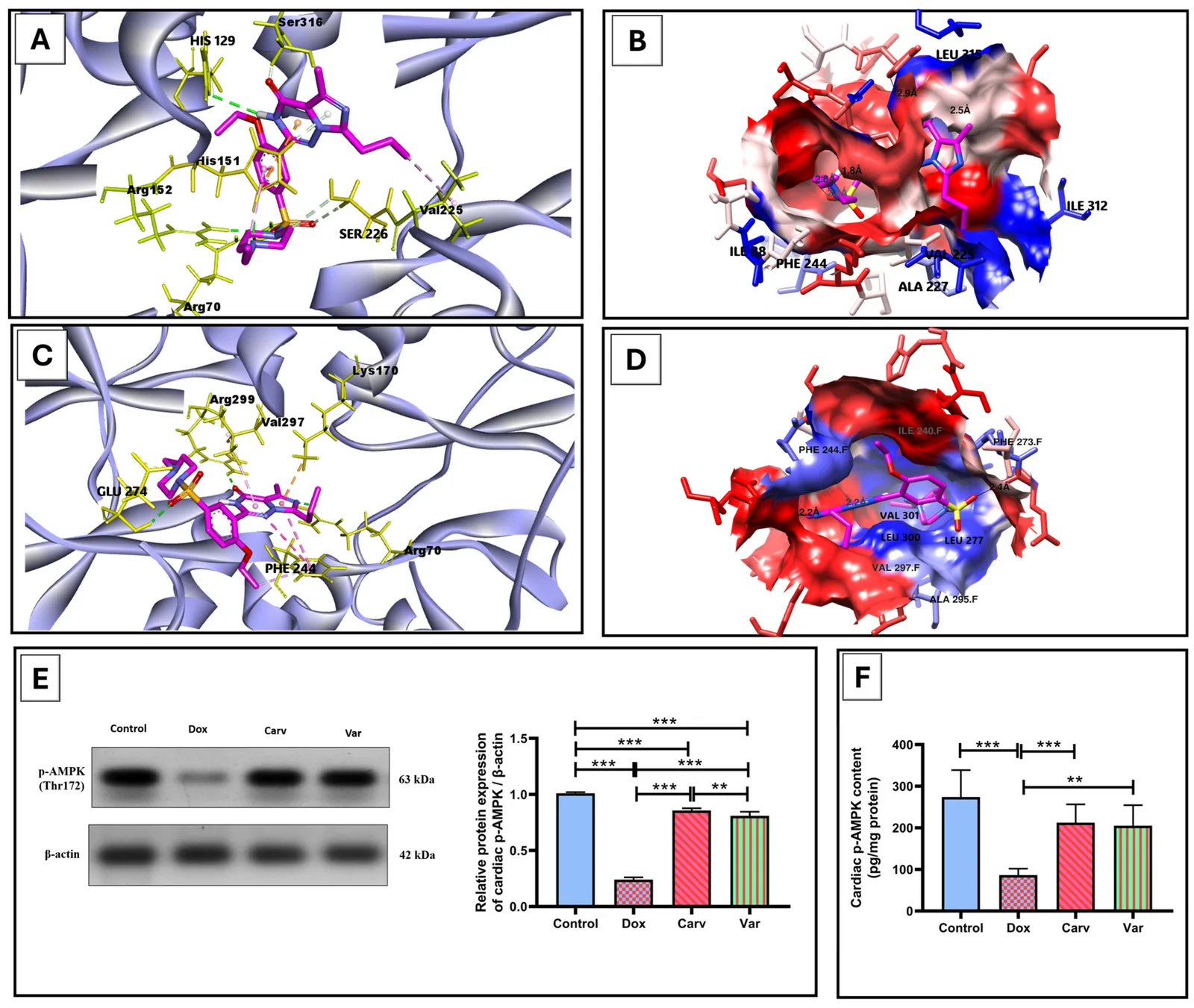
Regulation of AMPK activity by Var. The interaction of Var with AMPK was examined through molecular docking and protein expression analysis. Docking simulations were performed by fitting Var into the binding pockets of AMPK chains E and F, followed by evaluation of AMPK protein expression levels. (**A**) Amino acid residues involved in the interaction within AMPK chain E, visualized using Discovery Studio Visualizer. (**B**) Hydrophobic interactions between Var and the binding pocket of AMPK chain E, visualized with Chimera. (**C**) Amino acid residues contributing to the interaction within AMPK chain F, visualized using Discovery Studio Visualizer. (**D**) Hydrophobic contacts between Var and the binding site of AMPK chain F, visualized with Chimera. (**E**) Western blot analysis of AMPK protein expression across experimental groups (*n* = 3 biological replicates per group, where each biological replicate represents one animal). (**F**) Level of cardiac p-AMPK protein (pg/mg protein) by ELISA (*n* = 6 biological replicates per group, where each biological replicate represents one animal). Data are presented as mean ± SD. Statistical significance denoted as *** *p* ≤ 0.001, ** *p* ≤ 0.01.

To clarify the underlying mechanisms by which Var alleviated DIC, a docking study was carried out to investigate the possible binding affinity of Var toward the AMPK protein. Autodock Vina 1.1.2 was used to assess binding interactions of Var/AMPK [39]. The binding affinity was −4.1 Kcal/mol when Var interacted with the AMPK at the C2Z site. Var formed four hydrogen bonds with HIS151, HIS169, ARG70 and ARG152 of AMPK chain E. Hydrophobic interactions with ILE312, VAL225, ILE88, LEU315, ILE146, ILE150, PHE244, and ALA227 facilitate Var’s binding with AMPK. When Var interacted with the AMPK at the AMP site, the binding affinity was −5.3 Kcal/mol. Var formed three hydrogen bonds with ARG70, ARG299 and GLU 274 of AMPK chain F. Many hydrophobic interactions involving PHE244, ILE240, PHE273, VAL276, LEU277, VAL301, LEU300, VAL297, and ALA295 facilitate Var’s binding with AMPK (Figure 4A–D).

Western blot analysis of myocardial tissue revealed a significant 76.24% decrease in p-AMPK protein expression (F (3, 8) = 610.6, *p* < 0.0001, *n* = 3) compared with the control group. While Carv and Var treatment increased myocardial protein expression of p-AMPK by 3.57-fold (F (3, 8) = 610.6, *p* < 0.0001, *n* = 3) and 3.38-fold (F (3, 8) = 610.6, *p* < 0.0001, *n* = 3), respectively, compared with the Dox group. In addition, Carv treatment increased p-AMPK protein expression by 5.4% (F (3, 8) = 610.6, *p* < 0.01, *n* = 3) compared with the Var group (Figure 4E).

In addition, ELISA analysis of myocardial tissue revealed a significant 68.4% decrease in p-AMPK content (F (3, 20) = 16.72, *p* < 0.0001, *n* = 6) compared with the control group. While Carv and Var treatment increased myocardial p-AMPK level by 145.1% (F (3, 20) = 16.72, *p* < 0.001, *n* = 6) and 136.7% (F (3, 20) = 16.72, *p* < 0.01, *n* = 6), respectively, compared with the Dox group. These results indicate that both Carv and Var effectively counteracted the Dox-induced suppression of p-AMPK, suggesting a potential role for AMPK activation in their cardioprotective mechanisms (Figure 4F).

### 3.3. Var Upregulated SIRT1 and PPARγ in Dox-Treated Rats

**Figure 5:**
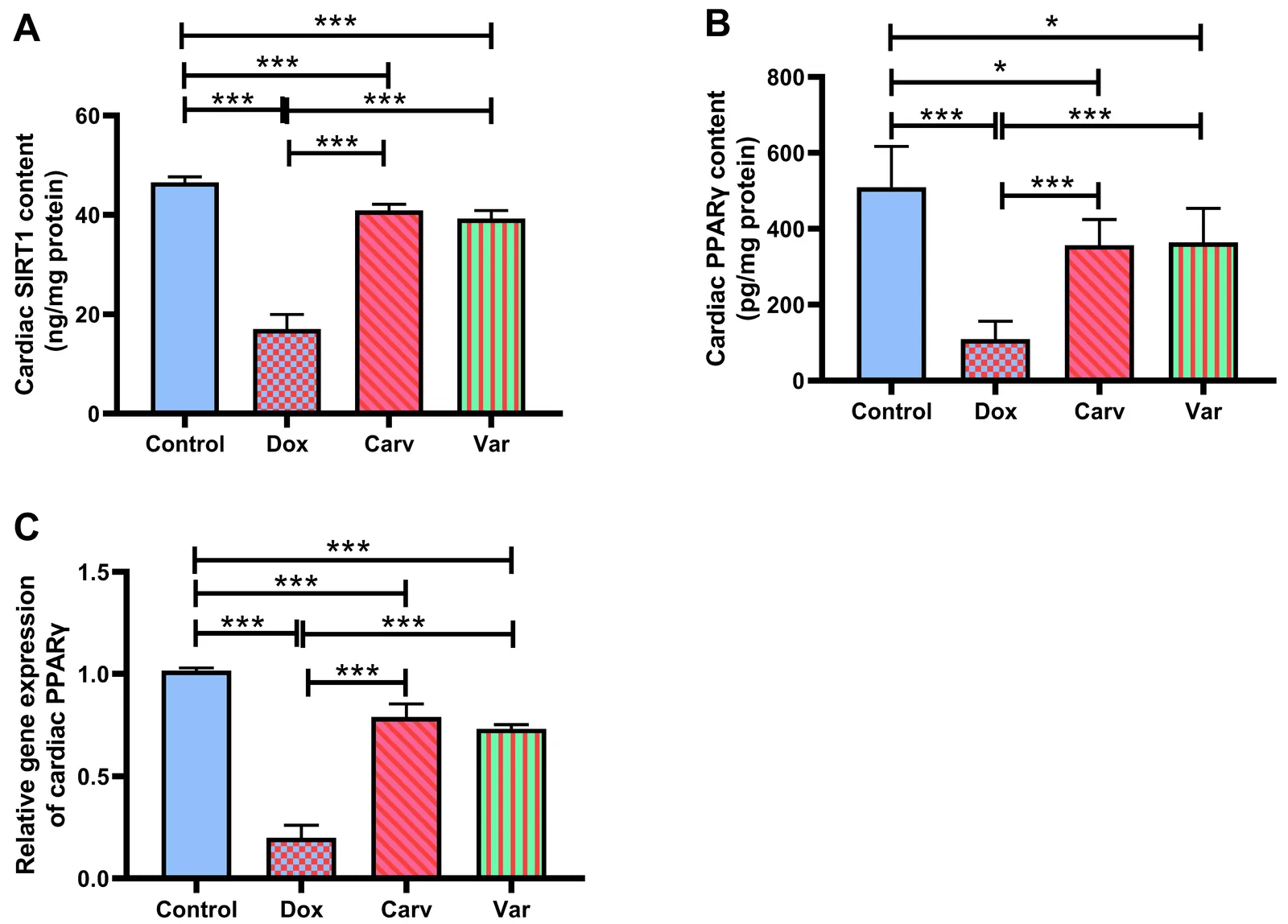
Var upregulated SIRT1 and PPARγ in Dox-treated rats. (**A**) The levels of cardiac SIRT1 (ng/mg protein) via ELISA. (**B**) The levels of cardiac PPARγ (pg/mg protein) via ELISA. (**C**) Cardiac PPARγ mRNA expression was determined by qRT-PCR. Data are presented as mean ± SD (*n* = 6 biological replicates per group, where each biological replicate represents one animal). Statistical significance denoted as * *p*  < 0.05, and *** *p* ≤ 0.001.

Activation of SIRT1 and PPARγ has been shown to restore cardiac function in models of Dox-induced cardiotoxicity [40,41]. To determine whether Var exerts similar effects in our experimental model, myocardial SIRT1 and PPARγ protein levels were quantified using ELISA, while PPARγ gene expression was evaluated by RT-qPCR. As illustrated in Figure 5A, Dox administration markedly reduced myocardial SIRT1 content by 63.5% (F (3, 20) = 280.4, *p* < 0.0001, *n* = 6), compared with the control group. Carv increased myocardial Sirt1 content by 2.41-fold (F (3, 20) = 280.4, *p* < 0.0001, *n* = 6) when compared with the Dox group. Var increased myocardial Sirt1 content by 2.31-fold (F (3, 20) = 280.4, *p* < 0.0001, *n* = 6) when compared with the Dox group.

In addition, Dox administration markedly reduced myocardial PPARγ content and PPARγ mRNA expression by 78.5% (F (3, 20) = 25.19, *p* < 0.0001, *n* = 6) and 80.33% (F (3, 20) = 345.9, *p* < 0.0001, *n* = 6), respectively, compared with the control group. Carv increased myocardial PPARγ content and gene expression of PPAR-γ by 226.8% (F (3, 20) = 25.19, *p* < 0.001, *n* = 6) and 3.95-fold (F (3, 20) = 345.9, *p* < 0.0001, *n* = 6), respectively, when compared with the Dox group. Var increased myocardial PPARγ content and gene expression of PPAR-γ by 233.5% (F (3, 20) = 25.19, *p* < 0.001, *n* = 6) and 3.67-fold (F (3, 20) = 345.9, *p* < 0.0001, *n* = 6), respectively, when compared with the Dox group (Figure 5B,C). These findings suggest that both Carv and Var activate the SIRT1/PPARγ signaling axis, thereby potentially contributing to their cardioprotective actions against Dox-induced myocardial injury.

### 3.4. Var Attenuated Dox-Induced Oxidative Stress and Modulated Nrf2/HO-1 Signaling Pathway

**Figure 6:**
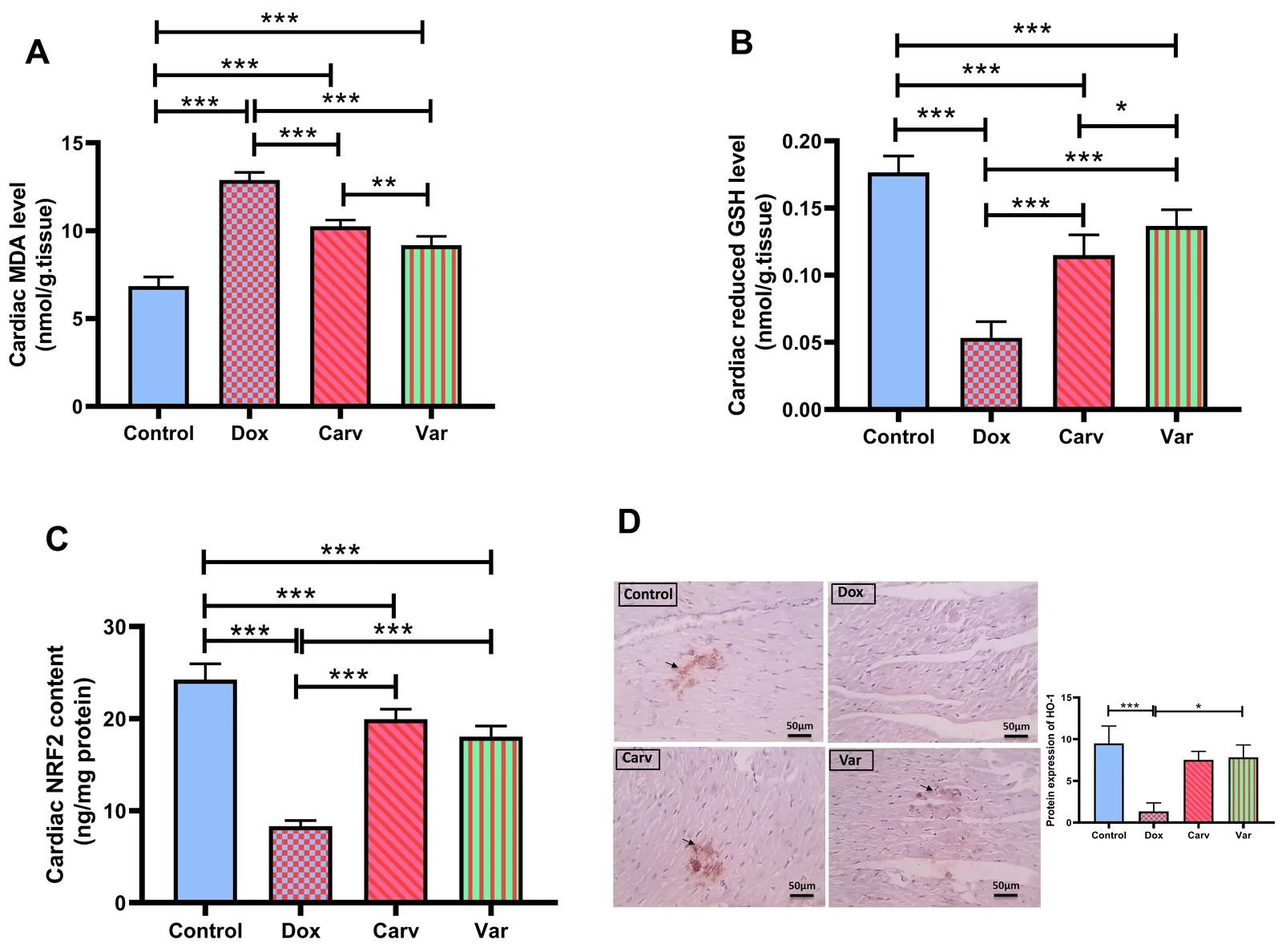
Var attenuated Dox-induced oxidative stress and modulated Nrf2/HO-1 signaling pathway. The antioxidant activity of Var was assessed by measuring: (**A**) level of cardiac MDA (nmol/g. tissue) spectrophotometry, (**B**) level of cardiac GSH (nmol/g. tissue) spectrophotometry, (**C**) level of cardiac Nrf2 protein (ng/mg protein) by ELISA, (**D**) level of HO-1 protein by immunohistochemistry. Positive immunoreactivity is indicated by black arrows. Tissue sections were counterstained with Mayer’s hematoxylin. Data are presented as Mean ± SD (*n* = 6 biological replicates per group, where each biological replicate represents one animal), with statistical significance denoted as (*** *p* ≤ 0.001, ** *p* ≤ 0.01, and * *p* < 0.05).

As illustrated in Figure 6**,** induction of cardiotoxicity with Dox resulted in a significant elevation of myocardial MDA content and reduced GSH level by 1.88-fold (F (3, 20) = 182.8, *p* < 0.0001, *n* = 6) and 69.82% (F (3, 20) = 95.02, *p* < 0.0001, *n* = 6), respectively, when compared with the control group. Carv treatment in Dox-intoxicated rats significantly decreased myocardial MDA content and increased GSH level by 20.48% (F (3, 20) = 182.8, *p* < 0.0001, *n* = 6) and 2.16-fold (F (3, 20) = 95.02, *p* < 0.0001, *n* = 6), respectively, when compared with the Dox group. Var treatment decreased myocardial MDA content and increased GSH level by 28.76% (F (3, 20) = 182.8, *p* < 0.0001, *n* = 6) and 2.56-fold (F (3, 20) = 95.02, *p* < 0.0001, *n* = 6), respectively, when compared with the Dox group. Also, Var treatment decreased myocardial MDA content and increased GSH level by 10.4% (F (3, 20) = 182.8, *p* < 0.01, *n* = 6) and 18.8% (F (3, 20) = 95.02, *p* < 0.0001, *n* = 6), respectively, when compared with the Carv group, as can be seen in Figure 6A,B.

Moreover, Dox treatment markedly downregulated myocardial Nrf2 and HO-1 protein levels by 65.66% (F (3, 20) = 188.8, *p* < 0.0001, *n* = 6) and 85.97% (*p* = 0.0008, *n* = 6), respectively, compared with the control group. Conversely, Carv increased myocardial Nrf2 and HO-1 levels by 2.4-fold (F (3, 20) = 188.8, *p* < 0.0001, *n* = 6) and 5.63-fold (*p* = 0.0955, *n* = 6), respectively, relative to Dox-treated rats. Similarly, Var increased reduced myocardial Nrf2 and HO-1 levels by 2.17-fold (F (3, 20) = 188.8, *p* < 0.0001, *n* = 6) and 5.88-fold (*p* = 0.0433, *n* = 6), respectively, compared with the Dox group. Collectively, these results suggest that Var mitigates Dox-induced oxidative damage by enhancing endogenous antioxidant defenses through augmenting antioxidant capacity, as seen in Figure 6C,D.

### 3.5. Var Mitigated Dox-Triggered Fibrosis and Apoptosis in DIC Rat Model

**Figure 7:**
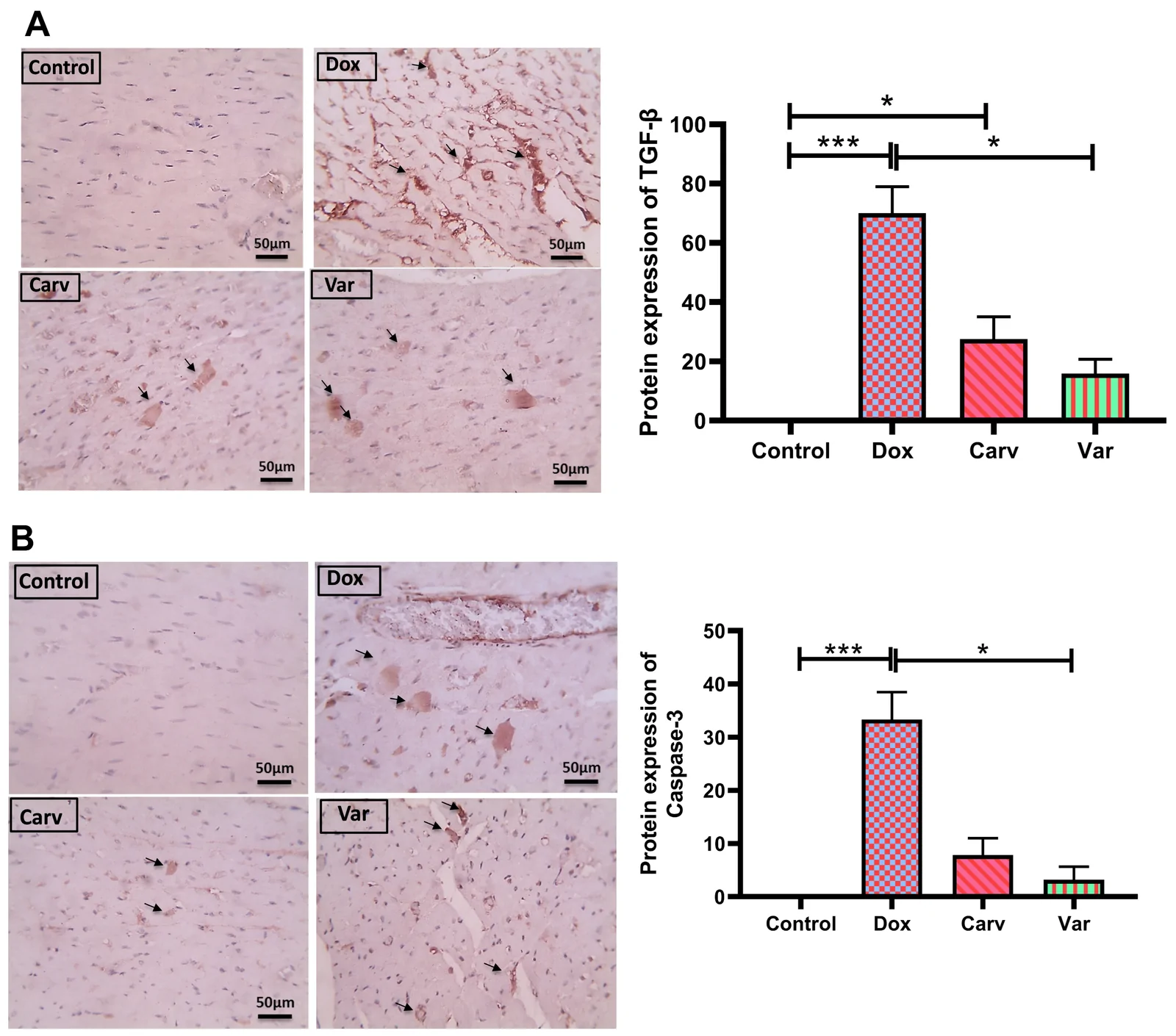
Var mitigated Dox-triggered fibrosis and apoptosis in the DIC rat model. Antifibrotic and anti-apoptotic activities of Var in Dox-treated rats were evaluated by the measurement of: (**A**) TGF-β protein expression by immunohistochemistry, (**B**) Caspase-3 protein expression by immunohistochemistry. Positive immunoreactivity is indicated by black arrows. Sections were counterstained with Mayer’s hematoxylin. Data are presented as mean ± SD (*n* = 6 biological replicates per group, where each biological replicate represents one animal). Statistical significance represented as *** *p* ≤ 0.001, and * *p* < 0.05, scale bar 50 µm.

In the Dox-treated group, myocardial TGF-β1 protein expression was markedly elevated, approximately 70-fold higher than that observed in the control group (*p* < 0.0001, *n* = 6), whereas Var administration resulted in a more substantial reduction of 77.39% (*p* = 0.0259, *n* = 6) compared with the Dox group (Figure 7A).

In the Dox group, our results demonstrated a 33.33-fold elevation in myocardial protein expression of Caspase-3 (*p* < 0.0001, *n* = 6) when compared with the control group. Var decreased myocardial protein expression of Caspase-3 by 90.5% (*p* = 0.0244, *n* = 6) when compared with the Dox group (Figure 7B).

## 4. Discussion

Dox has long been a cornerstone in chemotherapeutic regimens for various cancers, but its cardiotoxic effects pose significant challenges. The prevailing mechanism underlying Dox-induced cardiomyopathy involves the overproduction of reactive oxygen species (ROS). Consequently, the use of cardioprotective agents has emerged as a crucial strategy to alleviate Dox-associated cardiac injury [42]. Phosphodiesterase-5 (PDE-5) inhibitors have gained attention as potential therapeutics for cardiovascular disorders [43]. Numerous preclinical and clinical investigations have demonstrated the protective cardiovascular effects of sildenafil and similar PDE-5 inhibitors [44,45]. Notably, a clinical trial reported that sildenafil effectively prevented the deterioration of cardiac function in patients with preserved ejection fraction. In the current study, we provide findings indicating that Var, a PDE-5 inhibitor, exhibits protective activity against Dox-induced cardiac injury.

The administration of Dox in cancer therapy is well known to cause pronounced cardiotoxic effects [46,47]. Dox-induced cardiotoxicity typically manifests as body weight loss, increased serum activities of lactate dehydrogenase (LDH) and creatine kinase-MB (CK-MB), and left ventricular dysfunction [48,49,50]. Serum LDH and CK levels serve as key biomarkers of myocardial injury and are commonly used to assess cardiac toxicity [51]. During cardiovascular disease (CVD) or cardiac injury, CK-MB and LDH levels in heart tissue are markedly elevated [52,53,54]. In the present study, we preliminarily evaluated the cardiotoxic effects of Dox by measuring cardiac diagnostic markers. Our findings demonstrate that Dox-treated rats exhibited significant body weight loss and cardiac dysfunction relative to controls. Additionally, Dox exposure led to increased LDH leakage, indicative of cellular necrosis [55], and elevated serum CK and CK-MB levels, suggesting severe cardiomyocyte damage. These results align with previous findings reported by Fisher et al. [56]. Notably, treatment with the PDE-5 inhibitor Var significantly reduced these biomarkers, indicating cardioprotective effects. Furthermore, histopathological analysis revealed substantial cardiac tissue damage following Dox exposure, whereas Var treatment mitigated these pathological changes. Consistent with prior studies, the cardioprotective activity of Var has also been reported in the model of epinephrine-induced arrhythmia [34].

The present protocol represents a subacute (early-stage) model of Dox-induced cardiotoxicity, characterized by repeated administration of a cumulative dose of 15 mg/kg over two weeks with evaluation at four weeks. Anthracycline cardiotoxicity may be acute or chronic depending on the onset of the first symptomatic manifestations [57]. Acute cardiotoxicity usually occurs within the first few days of anthracycline administration and resembles acute myocarditis with myocyte damage, inflammatory infiltrates, and interstitial edema [58]. Clinically, acute anthracycline cardiotoxicity manifests with electrocardiographic changes (20–30%), neutropenia [57], arrhythmias (3%), and, in some patients, reversible cardiac dysfunction [59].

The concurrent reductions in IVSd, IVSs, and LVIDd observed in the doxorubicin-treated group likely reflect the early structural remodeling that occurs during the subacute phase of anthracycline cardiotoxicity. Acute doxorubicin injury is characterized by oxidative stress, inflammatory responses, myocardial edema [60], cardiomyocyte apoptosis, and myofibrillar degeneration [61]. Moreover, genetic deletion of p53 in mice protected against early anthracycline-induced cardiac dysfunction two weeks following doxorubicin exposure [62], highlighting the importance of early injury pathways in anthracycline cardiotoxicity. The combined effects of these pathological processes may reduce both ventricular wall thickness and left ventricular chamber dimensions during the subacute stage, before the ventricular dilation typically observed in chronic anthracycline cardiomyopathy. This interpretation is supported by previous experimental studies demonstrating reduced cardiac mass together with decreased LVIDd following doxorubicin administration [63]. Importantly, prophylactic administration of Carv or Var markedly attenuated these structural and functional abnormalities, preserving ventricular geometry and systolic performance, as evidenced by the restoration of IVSd, IVSs, LVIDd, EF%, and FS% toward control values.

AMPK serves as a fundamental cellular energy sensor, playing a crucial role in maintaining metabolic homeostasis in mammalian cells. It has been shown to confer protection against cardiac hypertrophy [64,65,66] and exert beneficial effects in heart failure [11,67,68,69]. Structurally, AMPK is a heterotrimeric complex comprising a catalytic α-subunit and two regulatory subunits, β and γ [70]. Among the two α-subunit isoforms, α1 is predominantly expressed in endothelial cells, while α2 is more abundant in cardiomyocytes [70,71]. Phosphorylation at Thr^172^ of the α-subunit significantly enhances AMPK activity, thereby regulating energy balance through direct phosphorylation of downstream targets or transcriptional modulation of energy-related genes [64,70,71]. Studies utilizing AMPK-deficient mouse models and pharmacological activators such as AICAR and metformin have demonstrated the cardioprotective role of AMPK against various cardiac pathologies, including diabetic cardiomyopathy [72], myocardial ischemic injury [73,74], heart failure [75], and pathological cardiac remodeling [76]. While reduced AMPK activity is a characteristic hallmark of myocardial ischemia and heart failure [73,77], its downregulation has also been recognized as a detrimental consequence of Dox treatment [78,79,80]. PDE-5 specifically degrades cyclic guanosine monophosphate (cGMP), and its inhibition enhances cGMP availability [81]. The cGMP/protein kinase G (PKG) signaling cascade has been identified as an upstream regulator of AMPK [82]. Research by Deshmukh et al. demonstrated that elevated cGMP levels stimulate glucose uptake in skeletal muscle cells in conjunction with AMPK activation, an effect abolished by guanylate cyclase inhibition [83]. In this study, we observed that treatment with the PDE-5 inhibitor Var significantly restored p-AMPK expression levels in cardiac tissues following Dox exposure. These observations are consistent with the findings of Weng et al. (2024), who reported that TP-10, a selective PDE-10A inhibitor, mitigated cardiac hypertrophy and fibrosis through the cAMP/AMPK and cGMP/PKG signaling pathways, respectively [84].

AMPK plays a pivotal role in modulating the activity of Sirtuin 1 (SIRT1) by increasing intracellular NAD^+^ concentrations, thereby activating the NAD^+^-dependent SIRT1 pathway to mediate various biological effects [15,85]. Conversely, SIRT1 overexpression has been shown to enhance the expression of liver kinase B1 (LKB1), a key upstream regulator of AMPK, leading to increased AMPK activation [86]. Collectively, AMPK and SIRT1 cooperatively regulate the expression of peroxisome proliferator-activated receptor gamma coactivator 1-alpha (PGC-1α), a key molecule responsible for protecting cells from oxidative stress [87,88]. Disruptions in this pathway have been observed in pathological conditions. For example, podophyllotoxin-induced SIRT1 downregulation leads to reduced LKB1 deacetylation, increasing acetylated LKB1 levels and thereby impairing AMPK phosphorylation. The resulting inhibition of AMPK activity decreases the expression of downstream targets such as PGC-1α and peroxisome proliferator-activated receptor (PPAR), ultimately leading to dysregulation of cardiac energy metabolism [89]. PGC-1α functions as a transcriptional coactivator of PPARγ [90,91], a member of the nuclear hormone receptor superfamily that, despite its relatively low expression in the heart, plays a crucial role in regulating cardiovascular physiology [92,93,94,95]. In addition, PPARγ has been implicated in cardiac disease, with studies demonstrating that cardiomyocyte-specific PPARγ knockout models exhibit spontaneous hypertrophic remodeling [96,97], while PPARγ activation has been shown to counteract hypertrophic signaling in response to pathological stimuli [98]. Notably, PPARγ activation has been reported to mitigate Dox- mediated cardiotoxic injury by reducing oxidative stress and inflammation in cardiomyocytes [99]. Conversely, PPARγ inhibition or deletion has been associated with increased vulnerability to oxidative stress.

Several compounds have been shown to exert cardioprotective effects via PPARγ activation. For example, Yan et al. demonstrated that piperine alleviates oxidative stress, inflammation, and programmed cell death in a Dox-induced cardiotoxicity model through PPARγ activation [100]. Similarly, L-carnitine has been shown to protect against imatinib-mediated cardiac injury by upregulating PPARγ [101], while atorvastatin, a long-acting statin, has been found to confer cardioprotective effects in paraquat-exposed models by enhancing PPARγ activity [102]. PPARγ activation also modulates inflammatory heart diseases by upregulating inhibitors of kappa B (IκB), thereby preventing NF-κB nuclear translocation and reducing inflammatory cytokine production [103]. Conversely, the downregulation of PPARγ has been implicated as a potential mechanism underlying Dox-induced cardiotoxicity [99,104]. In agreement with previous research, our study demonstrated that Dox exposure significantly decreased SIRT1 protein levels and PPARγ mRNA expression. However, treatment with the PDE-5 inhibitor Var effectively restored SIRT1 and PPARγ levels while suppressing MDA production, thereby mitigating oxidative stress.

Nrf2 serves as a master transcription factor that orchestrates the cellular defense mechanism against oxidative and toxic stress by activating genes involved in antioxidant responses and drug detoxification [105,106]. Activation of Nrf2 is tightly regulated by AMPK, which phosphorylates Nrf2 at the Ser550 site and concurrently inhibits glycogen synthase kinase 3β (GSK3β). These combined actions facilitate Nrf2 nuclear translocation and the initiation of antioxidant response element (ARE)-dependent gene expression [107]. Evidence indicates that deletion of Nrf2 aggravates Dox-induced myocardial injury [108]. The signaling cascade mediated by AMPK and SIRT1 is also critical in mitigating oxidative stress and inflammation by suppressing ROS production and inflammatory cytokines [109]. Inhibition of SIRT1 using pharmacological agents has been associated with elevated ROS levels, highlighting the direct link between SIRT1 and oxidative stress regulation [110]. Previous studies have demonstrated that SIRT1 upregulation alleviates Dox-induced cardiotoxicity in vivo by modulating apoptosis and oxidative stress [111]. Additionally, transgenic overexpression of SIRT1 in mouse heart has been reported to provide protection against paraquat-induced oxidative stress [112], whereas SIRT1 depletion exacerbates ischemia/reperfusion-induced oxidative damage [113]. Furthermore, PPARγ contributes to redox regulation by repressing NF-κB signaling and promoting the expression of antioxidant enzymes [114]. In the current investigation, treatment with the PDE-5 inhibitor Var markedly increased cardiac Nrf2 expression in Dox-challenged rats, accompanied by elevated levels of its downstream antioxidant mediators, such as heme oxygenase-1 (HO-1) and glutathione (GSH) [115]. Taken together, our findings suggest that Var functions as an effective antioxidant, protecting cardiomyocytes from oxidative damage.

**Figure 8:**
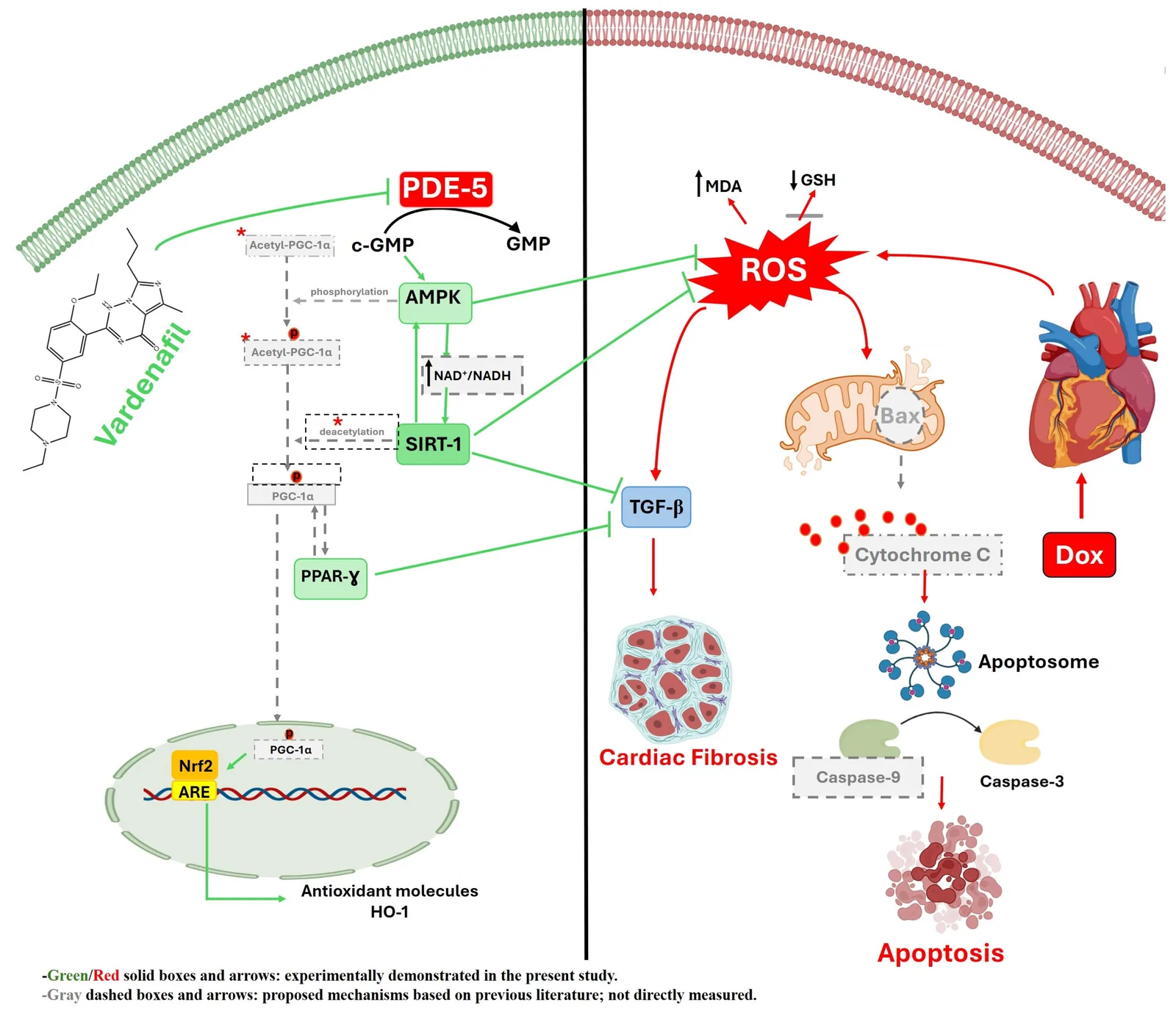
Proposed mechanism underlying the cardioprotective effects of vardenafil against doxorubicin-induced cardiotoxicity based on the present findings and published literature. Vardenafil treatment was associated with restoration of the p-AMPK/SIRT1 signaling pathway, accompanied by enhanced Nrf2 activation, upregulation of HO-1 and PPARγ, attenuation of oxidative stress, and reduced myocardial injury. The downstream effects on mitochondrial function and apoptosis represent a proposed mechanistic model derived from integration of the present findings with previous literature. Molecules enclosed within gray dashed boxes and marked with a red asterisk (*) (PGC-1α, Bax, cytochrome c, and caspase-9), together with their associated gray dashed arrows, were not experimentally evaluated in the present study and are included based on previously published evidence to illustrate potential downstream mechanisms. In contrast, green/red solid boxes and arrows represent experimentally measured findings obtained in the present study.

Elevated oxidative stress and impaired antioxidant defense mechanisms are key contributors to Dox-induced cardiac injury [116]. Dox promotes excessive intracellular ROS generation while simultaneously depleting endogenous antioxidant levels. Previous studies [111,116,117] have demonstrated that Dox exposure leads to increased MDA and lactate dehydrogenase (LDH) levels in mice, accompanied by a marked reduction in the activities of major antioxidant enzymes, including superoxide dismutase (SOD) and glutathione peroxidase (GSH-PX), thereby exacerbating oxidative stress-related damage. MDA serves as a key biomarker of lipid peroxidation, while GSH plays a critical role in neutralizing lipid peroxides by acting as a substrate for glutathione peroxidase 4 (GPx-4) [118]. The tissue levels of GSH reflect the overall antioxidant defense capacity. Consistent with previous findings, the results of this study indicate that Dox exposure leads to excessive ROS accumulation, resulting in a substantial depletion of GSH and an increase in lipid peroxidation, as evidenced by elevated MDA levels. These findings suggest that Dox-induced cardiotoxicity arises from augmented oxidative stress coupled with impairment of the endogenous antioxidant defense system (Figure 8).

TGF-β1 signaling is known to be closely modulated by both AMPK and SIRT1 [119,120]. Pharmacological activation of SIRT1 has been reported to inhibit cardiac fibroblast activation, potentially through modulation of the TGF-β/SMAD3 pathway. This aligns with previous findings demonstrating that SIRT1 suppresses TGF-β-mediated collagen production and myofibroblast differentiation [121,122]. Notably, Cappetta et al. [40] reported that resveratrol-mediated SIRT1 activation mitigates cardiac fibrosis by suppressing the TGF-β/SMAD3 signaling cascade. Additionally, SIRT1 is recognized as a crucial factor in preventing pathological cardiac remodeling and organ fibrogenesis [123]. An in vivo study has shown that cardiac tissues exposed to Dox exhibit elevated TGF-β expression, indicating a pro-fibrotic response. However, prophylactic administration of Var markedly reduced TGF-β levels in the hearts of Dox-treated rats, suggesting attenuation of the pro-fibrotic response induced by Dox. Given the concomitant increase in SIRT1 observed in the Var-treated group, these results support the concept that Dox promotes the expansion of a pro-fibrotic population of activated cardiac fibroblasts in vivo, while SIRT1 activation effectively reverses this pathological process [40]. Furthermore, the observed increase in fibrotic markers following Dox exposure in the present study may be attributed to excessive ROS production, which serves as a potent inducer of aberrant collagen synthesis and extracellular matrix (ECM) degradation.

Cardiomyocyte apoptosis is a major pathogenic mechanism underlying Dox-induced acute cardiotoxicity [79]. Among the various mechanisms implicated, excessive ROS generation in the myocardium is widely recognized as the primary trigger of Dox-induced cardiomyocyte apoptosis. This oxidative stress activates the intrinsic, mitochondria-dependent apoptotic pathway [124,125]. Previous studies have shown that ROS accumulation disrupts mitochondrial membrane potential (ΔΨm), induces the opening of the mitochondrial permeability transition pore (MPTP), and facilitates cytochrome c release—events that culminate in caspase-3 activation and DNA fragmentation, the hallmark features of apoptosis [126]. In addition, elevated ROS levels have been reported to upregulate TGF-β expression and promote its secretion [127]. The secretion of TGF-β1 from damaged cardiomyocytes has been implicated in apoptosis and hypertrophy [40,128]. Notably, previous studies have shown that PDE-5 inhibitors confer myocardial protection by inhibiting programmed cell death under various pathological conditions [56,129,130]. To assess Dox-induced myocardial apoptosis, caspase-3 expression was analyzed using immunohistochemistry. Our findings indicate that Dox treatment induced marked apoptotic injury in the heart, as evidenced by elevated caspase-3 expression in Dox-treated rats. However, in rats treated with Var, caspase-3 levels were downregulated in heart tissue, suggesting that Var may attenuate cardiomyocyte apoptosis in Dox-treated rats.

## 5. Conclusions

In conclusion, our study demonstrates that Var attenuates Dox-induced cardiotoxicity, as evidenced by improved cardiac function, reduced oxidative stress and apoptosis, and preservation of myocardial architecture. These protective effects were accompanied by restoration of p-AMPK/SIRT1 signaling and downstream cytoprotective markers, suggesting the involvement of this pathway in the observed cardioprotection. These findings support further investigation of Var as a potential cardioprotective strategy against chemotherapy-induced cardiotoxicity. However, the present study has several limitations. First, although molecular docking suggested a potential interaction between Var and AMPK, these findings are computational and do not demonstrate direct target binding. Moreover, while p-AMPK (Thr172) was experimentally quantified to confirm AMPK activation, direct ligand–target engagement was not assessed. Future studies employing biophysical approaches, such as surface plasmon resonance (SPR), isothermal titration calorimetry (ITC), or the cellular thermal shift assay (CETSA), are warranted to validate this interaction. Second, Carv- and Var-alone groups were not included, as the study was designed to evaluate their cardioprotective efficacy against doxorubicin-induced cardiotoxicity rather than their intrinsic effects in healthy myocardium. Third, only a single dose of vardenafil and carvedilol was evaluated, based on previously validated experimental protocols. Consequently, the present study does not establish dose–response relationships or define the optimal therapeutic dose. Future studies incorporating multiple dose levels and pharmacokinetic/pharmacodynamic analyses would further refine dose optimization and enhance clinical translatability. Fourth, the study employed an established acute doxorubicin-induced cardiotoxicity model with cardiac assessment performed shortly after completion of doxorubicin administration. Therefore, the findings primarily reflect protection against early anthracycline-induced cardiac injury and do not address the persistence of cardioprotection, delayed cardiac remodeling, or outcomes under clinically relevant multi-cycle chemotherapy regimens. Fifth, Western blot analysis was performed using a limited number of biological replicates, serving as a confirmatory assay for p-AMPK expression. Nevertheless, these findings were consistent with the quantitative ELISA results obtained from all animals, supporting the reliability of the observed changes in AMPK activation. Finally, although Var restored AMPK/SIRT1 signaling and downstream molecular markers, the causal hierarchy of this pathway was not directly investigated. Future studies using pharmacological inhibition or genetic manipulation of AMPK are needed to confirm the mechanistic role of AMPK in mediating these protective effects.

## Figures and Tables

**Table 1:** Primer sequences, accession numbers, and product sizes of the analyzed genes used for quantitative RT-PCR in heart tissue.

Gene of Interest		Primer Sequence	Reference Sequence	Product Size
β-actin	F	5′-ACTCTGTGTGGATTGGTGGC-3′	NM_031144.3	152
β-actin	R	5′-AAGGGTGTAAAACGCAGCTCA-3′		
PPAR γ	F	5′-AGAAGGCTGCAGCGCTAAAT-3′	NM_001145366.1	292
PPAR γ	R	5′-TGGGTCAGCTCTTGTGAACG-3′		

## Data Availability

All data and materials in our study are available upon reasonable request.
